# 伯基特淋巴瘤诊断与治疗中国专家共识（2026年版）

**DOI:** 10.3760/cma.j.cn121090-20260410-00186

**Published:** 2026-07

**Authors:** 中华医学会血液学分会淋巴细胞疾病学组 中国临床肿瘤学会（CSCO）淋巴瘤专家委员会

## Abstract

伯基特淋巴瘤在成人淋巴瘤中所占比例不到5％，临床表现呈高度侵袭性。尽管剂量密集化疗改善了患者的预后，但伯基特淋巴瘤仍然是一个具有特异临床和生物学特征的淋巴瘤亚型，成年伯基特淋巴瘤患者的诊断、治疗和预后因素目前尚无指南和共识建议。为加强我国临床医师对伯基特淋巴瘤的认识，提高诊断及治疗水平，推动开展多中心临床研究，中华医学会血液学分会淋巴细胞疾病学组、中国临床肿瘤学会（CSCO）淋巴瘤专家委员会组织相关专家，结合国内外最新研究进展，讨论并形成本共识。

## Abstract (en)

Burkitt lymphoma accounts for less than 5％ of adult lymphomas and is clinically highly aggressive. Despite improved prognosis with dose-intensive therapy, Burkitt lymphoma remains a specific area of clinical and biological research, and there are currently no consensus recommendations regarding the diagnosis, treatment, and prognostic factors in adult patients. In order to strengthen the understanding of Burkitt lymphoma in our country, improve the diagnosis and treatment level, and help promote multi-center clinical research, the Lymphoid Disease Group, Chinese Society of Hematology, Chinese Medical Association and Lymphoma Expert Committee of Chinese Society of Clinical Oncology（CSCO）organized relevant experts to discuss and form this consensus on the basis of the relevant consensus on the diagnosis and treatment of Burkitt lymphoma, combined with the latest research progress both domestically and internationally.

伯基特淋巴瘤（Burkitt lymphoma，BL）是起源于生发中心的高度侵袭性B细胞淋巴瘤。该病的名称来源于爱尔兰外科医师Denis Burkitt，他在1958年描述了38名乌干达儿童，这些儿童面部肿瘤迅速增大，且几乎无一生存[1]。1964年，科学家在BL细胞系中发现EB病毒（Epstein-Barr virus，EBV），提示病毒在肿瘤发生中起促进作用[2]。后续研究发现，MYC基因易位是BL发生发展的关键分子[3]。

BL患者临床呈现高度侵袭性，常伴腹腔大包块、易累及中枢神经系统（CNS）等表现，预后不良。虽然剂量密集化疗可改善BL患者的预后，但关于BL成年患者的诊断、治疗和预后因素，目前尚无指南或共识建议。为加强我国临床医师对BL的认识，提高诊断及治疗水平，中华医学会血液学分会淋巴细胞疾病学组、中国临床肿瘤学会（CSCO）淋巴瘤专家委员会组织相关专家参考国内外相关诊疗共识，并结合国内外最新的研究进展，讨论并制定本共识以供临床参考。

一、共识制订过程

共识制定目的为加强我国临床医师对BL的认识，提高诊断及治疗水平，目标人群为BL患者及其照护者，以及相关医疗专业人员。本共识由中华医学会血液学分会淋巴细胞疾病学组及中国临床肿瘤学会（CSCO）淋巴瘤专家委员会发起制订，启动时间为2025年7月16日，定稿时间为2026年4月8日，工作组成员专业覆盖血液病学、病理学、肿瘤学等多个领域，所有工作组成员均填写了利益冲突声明表，均声明不存在与本共识相关的经济或非经济利益冲突。制订过程或推荐意见形成未受到资助影响。

本共识在国际实践指南注册与透明化平台（http://www.guidelines-registry.cn）完成注册，注册号：PREPARE-2025CN1022。共识内容涵盖BL的流行病学特征、预防策略、诊断标准、治疗原则及预后评估。证据检索与筛选按照人群、干预、对照、结局（People, Intervention, Control, Outcome, PICO）的原则，应用主题词和自由词的检索策略进行系统检索，检索范围包括：（1）PubMed、Embase、The Cochrane Library、Web of Science等英文数据库；（2）中国知网、维普、万方等中文数据库；（3）美国国家综合癌症网络（National Comprehensive Cancer Network，NCCN），中国临床肿瘤学会（CSCO）等权威学术团体发布的相关指南与共识，美国血液学会（ASH）年会、欧洲血液学协会（EHA）年会、国际恶性淋巴瘤会议（ICML）等会议摘要等。检索时限范围从建库至2025年7月16日，纳入系统综述、荟萃分析、随机对照试验（randomized control trial，RCT）、队列研究、病例对照研究、病例报告和指南/共识等。根据2009年牛津循证医学中心证据分级和推荐强度标准（Oxford Center for Evidence-Based Medicine，OCEBM）（表1）对筛选出的临床证据进行分级[4]。全体工作组成员通过在线问卷的形式，对临床问题及推荐意见进行投票，对有争议的推荐意见进行相应的修改并再次讨论，直至最终达成共识，从而提升推荐意见的科学性和可靠性。

**表1:** 2009年牛津循证医学中心证据分级和推荐强度标准（OCEBM）[4]

推荐强度	证据强度	描述
A	1a	同质性好的多项随机对照试验（RCT）的系统评价
	1b	单个RCT（置信区间小）
	1c	“全或无”的证据
B	2a	同质性好的队列研究的系统评价
	2b	单个队列研究（包括低质量RCT，如随访率<80％）
	2c	基于患者结局的研究
	3a	同质性好的病例对照研究的系统评价
	3b	单个病例对照研究
C	4	病例系列报道（包括低质量队列研究及病例对照研究）
D	5	专家意见或评论

二、流行病学

根据WHO血液淋巴肿瘤第五版分类（WHO-5HAEM）推荐，BL属于成熟B细胞肿瘤[5]。BL根据既往流行病学背景和地理位置分为3种流行病学亚型：地方性BL、非地方性/散发性BL、免疫缺陷相关BL[6]。考虑到EBV感染在BL的早期发病机制中起重要作用，WHO-5HAEM基于EBV感染情况将BL分为2种亚型：EBV阳性BL和EBV阴性BL。

李小秋等[7]对国内多中心10 002例淋巴瘤病例进行分析，我国BL在全部淋巴瘤亚型中所占比例约为1％，略低于欧美国家[6]。EBV阳性BL在北非地区的患病率约为75％，在美国约为20％，而亚洲地区尚无相关流行病学数据[8]。成人BL患者发病年龄呈双峰分布，2个发病高峰的中位年龄分别为40岁和75岁，男女比例约为3∶1，常累及骨髓及CNS。

BL侵袭性极高，对化疗较敏感，部分患者可治愈。Evens等[9]在2021年回顾分析了2009–2018年美国30个肿瘤中心641例BL患者的数据，3年无进展生存（PFS）率及总生存（OS）率分别为64％和70％。儿童BL的预后较成人BL好。根据美国SEER（Surveillance Epidemiology End Results）数据库数据，BL的5年疾病相关死亡率在20～59岁人群中约为50％，而在60岁以上人群中为75％[8]。

三、临床诊断步骤、病理诊断及鉴别诊断

本共识主要聚焦于“非地方性/散发性BL”的诊疗推荐。

（一）临床诊断步骤

**推荐意见：首选淋巴结活检、手术切除活检或粗针活检，以进行组织病理学、免疫组织化学（IHC）和荧光原位杂交（FISH）检查。**

BL是一种成熟的、高度侵袭性B细胞非霍奇金淋巴瘤，肿瘤生长迅速，大量细胞凋亡，甚至在治疗开始前可合并肿瘤溶解综合征（TLS）。临床高度怀疑BL［如乳酸脱氢酶（LDH）高于正常值上限、结外尤其是回盲部受累、腹腔大肿块（肿块最大直径≥7 cm）、临床分期晚期（Ⅲ或Ⅳ期）或疾病进展迅速］患者应尽快行穿刺活检。

为获取足够的肿瘤样本，首选淋巴结活检、手术切除活检或粗针活检，以进行组织病理学、IHC及FISH检查。建议对腹水或胸腔积液样本进行细胞学、流式细胞术、常规细胞遗传学或FISH检查，以确定BL的诊断。

（二）病理诊断

组织病理学上，BL有特征性的形态。肿瘤细胞形态较一致，中等大小，镶嵌状或铺路石样排列，细胞质边界呈方形，核圆形，染色质细团块状，有多个靠近中心位置的嗜碱性核仁，核分裂象多，“星空”现象明显。免疫表型方面，肿瘤细胞表达广谱B细胞标志物（CD20、CD79α、CD19和CD22等），CD10和BCL6阳性，MUM1阴性或部分肿瘤细胞异质性阳性，呈生发中心免疫表型。BCL2阴性或弱阳性，C-MYC高表达（>80％），Ki-67增殖指数常大于95％。遗传学改变为IG::MYC融合阳性，其中80％为t（8;14）（q24;q32）易位，导致MYC::IGH，t（8;22）（q24;q11）、t（2;8）（p12;q24）易位少见，分别导致MYC::IGL、MYC::IGK。是否存在非IG::MYC融合的BL目前尚不确定，首先要排除高级别B细胞淋巴瘤（HGBL）或弥漫大B细胞淋巴瘤（DLBCL），需谨慎诊断。根据EBV编码的小RNA原位杂交检测结果阳性或阴性，可将BL诊断为EBV阳性BL或EBV阴性BL。基于上述病理特点，WHO-5HAEM提出了BL必要的（essential）和理想的（desirable）诊断标准。其中必要的标准包括：肿瘤细胞形态单一、中等大小、常具有嗜碱性胞质和多个小核仁；CD20和CD10阳性，BCL2阴性或弱阳性，Ki-67增殖指数>95％，常有>80％肿瘤细胞强表达C-MYC，有MYC断裂或IG::MYC易位证据。理想的标准包括：“星空”现象和肿瘤细胞黏附性生长；BCL6和CD38阳性，TDT阴性；排除BCL2和BCL6易位。因此，BL的准确、规范诊断需整合形态学、免疫表型、EBV状态和分子遗传学检测。

（三）鉴别诊断

BL的鉴别诊断包括B淋巴母细胞淋巴瘤/白血病、伴MYC和BCL2重排的DLBCL/HGBL（主要见于成人）、伴11q异常的HGBL和非特指型HGBL，以及在活检标本中与儿童型滤泡性淋巴瘤（主要见于儿童）鉴别。B淋巴母细胞淋巴瘤/白血病可通过形态学特征（染色质细致、胞质稀少）、末端脱氧核苷酸转移酶（TDT）的强表达及其他未成熟标志物（如CD34）阳性与BL鉴别。存在MYC和BCL2重排（无论是否伴BCL6重排）可排除BL的诊断，此类病例应诊断为伴有MYC和BCL2重排的DLBCL/HGBL；而同时存在MYC和BCL6重排的病例则被归类为非特指型DLBCL或非特指型HGBL。对于组织病理学形态和免疫表型与BL相似的淋巴瘤，若缺乏MYC重排，应评估其是否存在与伴11q异常的HGBL相关的11q获得/缺失模式。一些非特指型HGBL病例具有与BL相似的组织学特征，部分病例还存在MYC基因重排，进一步增加了与BL的相似性。然而，非特指型HGBL通常缺乏CD10表达，且BCL2表达较强。

四、治疗前评估、疾病分期及预后分层

（一）治疗前评估

**推荐意见：治疗前评估需要包括全血细胞检查、肝肾功能、血清LDH、病原学检查（HIV、EBV）、聚合酶链反应（EBV）、PET-CT或全身增强CT、骨髓活检及颅脑磁共振成像（MR）（可疑CNS受累患者）。排除外周血有BL细胞的情况下，应尽早进行腰椎穿刺脑脊液检查。**

必要的检查包括：全身体格检查（尤其注意结节受累区域和肝、脾是否肿大）、体能状态、B症状；实验室检查包括全血细胞检查、血生化检查、血清LDH水平、病原学检查（乙型肝炎、丙型肝炎、HIV相关检测）、妊娠相关检测；影像学检查推荐PET-CT（优先推荐）或颈、胸、腹、盆腔增强CT检查；骨髓病理活检和骨髓细胞学检查，其中骨髓活检样本长度应在1.5 cm以上；其他检查包括心电图和心脏超声。

在无禁忌证情况下（如外周血可检测到BL细胞），应尽早进行脑脊液检查。检查项目应包括脑脊液常规、生化及流式细胞术检查以除外CNS受累[10]。

优先推荐采用PET-CT作为初诊BL患者分期及疗效评估的检查手段，推荐使用2014年Lugano修订版Ann Arbor分期系统进行疾病分期[11]。

（二）预后分层

**推荐意见：推荐通过BL国际预后指数（BL-IPI）评分判断BL患者的预后。**

目前常用的BL预后分层为BL-IPI评分，该预后模型的建立基于美国2009–2018年633例BL患者，并通过欧洲、加拿大及澳大利亚2004–2019年收治的457例患者进行验证。预后因素包括年龄≥40岁、美国东部肿瘤协作组体能状态评分（ECOG评分）≥2分，LDH>3倍正常值上限及CNS受累。低危（无危险因素）、中危（1个危险因素）及高危（≥2个危险因素）患者的3年PFS率分别为92％、72％和53％，3年OS率分别为96％、76％和59％[12]。后续该模型进一步被捷克及印度的回顾性数据证实具有良好的预后分层价值[13]–[14]。

除此之外，患者是否合并TP53基因DNA结合区突变或治疗后是否达到完全缓解（CR）均影响预后[14]–[15]。

五、治疗

**推荐意见：确诊BL的患者应尽快启动抗肿瘤治疗。抗肿瘤治疗前需采取积极支持治疗，防止TLS、肠道并发症（梗阻/穿孔）及感染等情况发生。**

（一）TLS的识别、预防和治疗

**推荐意见：在启动治疗前后需密切观察并尽早识别TLS高危BL患者。预防措施包括减瘤治疗、大量水化碱化及预防高尿酸血症。**

BL患者在启动治疗前后有较高风险发生TLS。TLS是由恶性肿瘤细胞短时间内大量崩解，释放细胞内物质引发的代谢紊乱综合征，临床主要表现为高钾血症、高磷血症、低钙血症和高尿酸血症，可引发急性肾衰竭、心律失常等致命并发症。临床需尽早识别TLS高危患者，加强预防和监测，一旦发现立即开始治疗。TLS的诊断标准及分级标准见表2及表3。

**表2:** 肿瘤溶解综合征的诊断标准^a^

实验室标准	临床标准
成人血液尿酸≥0.08 g/L（476 µmol/L）	急性肾损伤定义为肌酐水平>1.5倍正常值上限，肌酐水平增加≥0.003 g/L（26.5 µmol/L）或尿量<0.5 ml·kg^−1^·h^−1^持续6 h及以上
成人血清无机磷≥0.045 g/L（1.5 mmol/L）	可能或确认由高磷血症导致的心律失常或猝死
血钾≥6.0 mmol/L	可能或确认由高钾血症导致的心律失常或猝死
血清校正钙<0.07 g/L（1.75 mmol/L）^b^或离子钙<4.5×10^−2^ g/L（1.12 mmol/L）	可能或确认由低钙血症导致的心律失常、猝死、癫痫发作、神经肌肉兴奋性增高、低血压或心力衰竭

**注** ^a^判断是否符合诊断标准时需满足以下条件：维持充分补液，并使用1种或多种降尿酸药物；在治疗前3 d或治疗后7 d内，24 h内至少符合2项实验室标准和1项临床标准。^b^血清校正钙（mmol/L）＝血清总钙（mmol/L）+0.2×［40−白蛋白（g/L）］

**表3:** 肿瘤溶解综合征的分级标准

临床或实验室表现	0级	Ⅰ级	Ⅱ级	Ⅲ级	Ⅳ级	Ⅴ级^a^
实验室标准	无	有	有	有	有	NA
肌酐	<1.5倍ULN	1.5倍ULN	>1.5倍且≤3.0倍ULN	>3.0倍且≤6.0倍ULN	>6.0倍ULN	NA
心律失常	无	无需干预	需非紧急医疗干预	有症状且未得到完全控制或通过设备（如除颤器）得到控制	危及生命（如心律失常伴心力衰竭、低血压、晕厥或休克）	NA
癫痫发作	无	无	1次短暂全身性发作，1次或多次通过抗惊厥药物得到良好控制的癫痫发作，或不影响日常生活的偶尔局灶性运动性发作	癫痫发作伴意识改变，或接受药物干预的情况下仍有癫痫发作伴突破性全身性发作	长时间、反复或难以控制的任何类型癫痫发作（如癫痫持续状态或难治性癫痫）	NA

**注** ULN：正常值上限；NA：不适用；^a^Ⅴ级为致死性

TLS的高危因素包括LDH升高（高于正常值上限），大包块（最大直径≥7 cm），骨髓受累或白血病样BL，外周血可检测出BL细胞，进展期（Ⅲ～Ⅳ期），ECOG评分≥2分及肾功能异常（肌酐清除率<80 ml/min）。

当临床出现至少1个TLS高危因素时，应立即启动TLS的预防。对于高肿瘤负荷的患者，应予小剂量激素联合环磷酰胺（200～300 mg/m^2^）减瘤治疗。同时，主要的TLS预防手段包括大量水化及预防高尿酸血症[16]。水化是预防或逆转TLS的关键手段，建议每24 h静脉注射2～3 L/m^2^且不含钾的液体，维持尿量为每小时2 ml/kg。预防高尿酸血症的药物主要选择黄嘌呤氧化酶抑制剂（别嘌呤醇或非布司他）或重组尿酸氧化酶（拉布立酶）。别嘌呤醇或非布司他主要通过抑制尿酸生成降低血尿酸水平，对溶解已形成的尿酸盐晶体无效，应在治疗前48～72 h开始使用便于药物起效。两项随机对照试验比较了别嘌呤醇与非布司他，结果未显示任一药物具有优效性[17]–[18]。而拉布立酶可将尿酸转化为可溶的尿囊素，快速降低尿酸水平，可有效溶解尿酸盐晶体。但在预防或治疗TLS的背景下，拉布立酶并未明确降低肾脏并发症的发生风险[19]。传统观点认为碱化治疗可提高尿酸溶解度并减少肾结晶沉积，但随着拉布立酶等高尿酸血症有效治疗方案的出现，及碱化治疗抑制磷排泄，可能加剧高磷血症，进而增加肾脏磷酸钙沉积风险或加剧低钙血症，导致手足搐搦、癫痫发作或心律失常风险增加，因此，对于拉布立酶可及的医疗中心，不再推荐碱化治疗。对于选择黄嘌呤氧化酶抑制剂进行高尿酸血症预防的中心，予口服/静脉碳酸氢钠碱化治疗维持尿液pH值在7.0以上。

对于诊断TLS的患者，应密切监测患者生命体征及实验室检查结果。高钾血症需紧急干预，治疗方案包括静脉注射钙剂（以拮抗钾离子对细胞膜的作用）及促进钾离子向细胞内流动的疗法（如胰岛素联合葡萄糖）。对于肾功能正常或轻度障碍患者，可应用袢利尿药物增加肾脏的钾排泄量。阳离子交换剂（如锆硅酸钠）可在胃肠道结合钾离子，促进其清除。对于有临床意义高钾血症、高磷血症、有症状低钙血症合并急性肾损伤患者，考虑积极进行肾脏替代治疗。

（二）初诊BL的治疗

**推荐意见：针对BL的初诊治疗选择主要通过患者的“治疗危险度”分级制定诊疗方案，方案以利妥昔单抗联合短疗程强化疗为主，都应包含CNS预防策略。**

针对BL的初诊治疗选择主要通过患者的“治疗危险度”分级制定，用于指导一线免疫化疗方案选择与强度。肿瘤EBV状态不影响治疗方案选择，EBV阳性及EBV阴性BL的初诊推荐治疗方案一致（图1）[6]。治疗危险度定义：低危为患者初诊时Lugano 2014疾病分期为Ⅰ期且单个、非腹腔包块直径<10 cm且LDH水平正常；治疗危险度高危指患者为Ⅰ期合并腹腔包块，或非腹腔包块直径>10 cm，或疾病分期为Ⅱ～Ⅳ期。

**图1:**
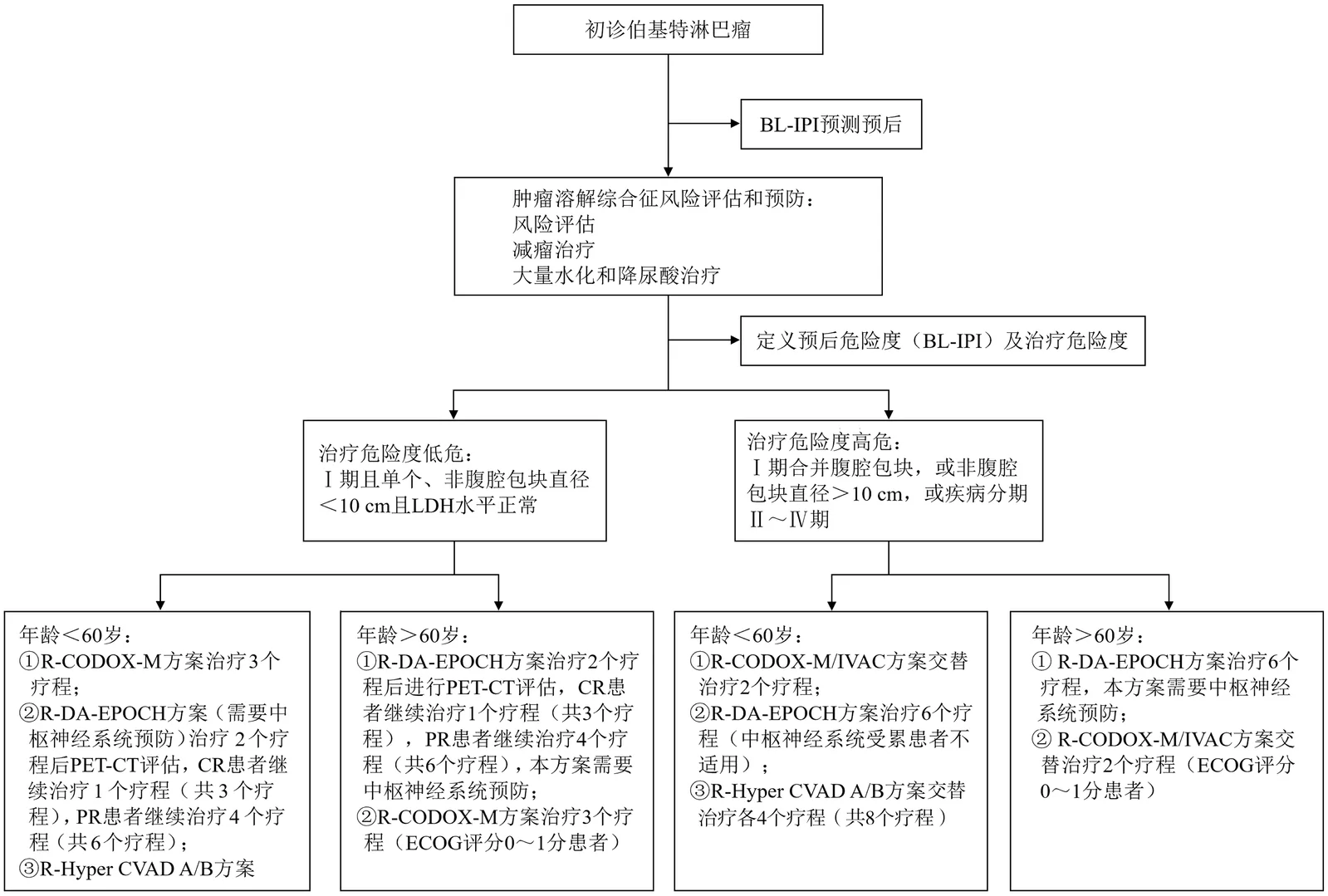
初诊伯基特淋巴瘤患者治疗流程图 **注** BL-IPI：伯基特淋巴瘤国际预后指数；R-CODOX-M：利妥昔单抗+环磷酰胺+长春新碱+多柔比星+甲氨蝶呤；R-DA-EPOCH：利妥昔单抗+依托泊苷+多柔比星+长春新碱+环磷酰胺+泼尼松；PET-CT：正电子发射计算机断层显像；CR：完全缓解；PR：部分缓解；R-Hyper-CVAD：A方案为利妥昔单抗+环磷酰胺+长春新碱+多柔比星+地塞米松，B方案为利妥昔单抗+甲氨蝶呤+阿糖胞苷；ECOG评分：美国东部肿瘤协作组体能状态评分；IVAC：异环磷酰胺+依托泊苷+阿糖胞苷

目前初诊BL的推荐治疗方案均以利妥昔单抗联合短疗程强化疗（CODOX-M/IVAC、EA-EPOCH或hyper CVAD A/B方案）为主（推荐强度A，证据强度1b）。2016年，法国开展的针对260例BL的Ⅲ期随机对照研究显示，中位随访38个月，利妥昔单抗的加入可以显著提高BL患者的3年无事件生存（EFS）率（75％对62％，*P*＝0.024）[20]，推荐BL患者初诊治疗联合利妥昔单抗。2023年，欧洲多中心的Ⅲ期随机对照研究在高危BL患者中对比R-CODOX-M/IVAC和R-DA-EPOCH方案的疗效。由于患者入组缓慢，该研究提前结束。研究共纳入89例BL患者，中位随访28.5个月，R-CODOX-M/R-IVAC方案组的2年PFS率为76％（95％ *CI*：60％～86％），R-DA-EPOCH方案组为70％（95％ *CI*：54％～82％），差异无统计学意义（*HR*＝1.42，95％ *CI*：0.63～3.18，*P*＝0.40）。虽然试验提前终止，但现有数据表明R-DA-EPOCH方案与R-CODOX-M/R-IVAC方案的PFS相似，但其安全性高且支持性需求较少[21]。

基于上述循证证据，本共识推荐根据患者年龄及治疗危险度选择治疗方案（各方案详细剂量见表4）（推荐强度A，证据强度1b）。

**表4:** 初诊伯基特淋巴瘤患者化疗方案剂量推荐

方案名称	周期	药物剂量
R-DA-EPOCH方案[22]	21 d	第1个疗程： 利妥昔单抗375 mg/m^2^ d0，依托泊苷50 mg/m^2^ d1～4（96 h连续输注），多柔比星10 mg/m^2^ d1～4（96 h连续输注），长春新碱0.4 mg/m^2^（最大剂量0.5 mg）d1～4（96 h连续输注），环磷酰胺750 mg/ m^2^ d5；泼尼松60 mg/m^2^ d1～5； 后续疗程根据前1个疗程中性粒细胞绝对计数（ANC）/血小板计数（PLT）最低值动态调整化疗药物剂量： ①环磷酰胺、多柔比星、依托泊苷同步同比例上调/下调： ANC≥0.5×10^9^/L且PLT≥50×10^9^/L，下1个疗程剂量增加20％； ANC（0.20～0.49）×10^9^/L或PLT（25～49）×10^9^/L，下1个疗程剂量不变； ANC<0.2×10^9^/L或PLT<25×10^9^/L，下1个疗程剂量减少20％； ②长春新碱一般不上调，出现严重神经不良反应则减量或停用； ③泼尼松剂量通常不作调整； ④利妥昔单抗剂量一般不调整，出现严重输液反应或感染风险极高时可酌情延迟或减量
R-CODOX-M方案[21]	21 d	利妥昔单抗375 mg/m^2^ d1、9；环磷酰胺800 mg/m^2^ d1，200 mg/m^2^ d2～5；长春新碱1.5 mg/m^2^ d1、8；多柔比星40 mg/m^2^ d1；甲氨蝶呤3 000 mg/m^2^ d10
R-IVAC方案（高危患者需与R-CODOX-M方案交替）[21]	21 d	利妥昔单抗375 mg/m^2^ d3、7；异环磷酰胺1 500 mg/m^2^ d1～5；依托泊苷60 mg/m^2^ d1～5；阿糖胞苷2 000 mg/m^2^ d1～2
R-hyper-CVAD方案（A/B方案交替）[23]–[24]	21 d	**A方案：** 利妥昔单抗375 mg/m^2^ d0，环磷酰胺300 mg/m^2^每12 h 1次×6次（d1～3），长春新碱2 mg d4、11，多柔比星50 mg/m^2^ d4，地塞米松40 mg/d d1～4、d11～14；并需预防性鞘内注射 **B方案：** 利妥昔单抗375 mg/m^2^ d0；甲氨蝶呤1 000 mg/m^2^维持24 h d1；甲氨蝶呤结束输注后24 h开始应用亚叶酸钙15 mg/m^2^口服或静脉注射每6 h 1次，直至甲氨蝶呤浓度<0.1 µmol/L；阿糖胞苷3 000 mg/m^2^静脉输注2 h，每12 h 1次×4次，d2～3

1. 年龄<60岁，低危BL患者：①R-CODOX-M方案治疗3个疗程；②R-DA-EPOCH方案治疗2个疗程后PET-CT评估，CR患者继续治疗1个疗程（共3个疗程），部分缓解（PR）患者继续治疗4个疗程（共6个疗程）[22]；③R-Hyper CVAD A/B方案。

2. 年龄≥60岁，低危BL患者：①R-DA-EPOCH方案治疗2个疗程后PET-CT评估，CR患者继续治疗1个疗程（共3个疗程），PR患者继续治疗4个疗程（共6个疗程）；②ECOG评分≤1分患者可选择R-CODOX-M方案治疗3个疗程。

3. 年龄<60岁，高危BL患者：①R-CODOX-M/IVAC方案交替治疗各2个疗程（共4个疗程）[21]；②R-DA-EPOCH方案治疗6个疗程；③R-Hyper CVAD A/B方案交替治疗各4个疗程（共8个疗程）[23]–[24]。

4. 年龄≥60岁，高危BL患者：①R-DA-EPOCH方案治疗6个疗程；②ECOG评分≤1分患者，可选R-CODOX-M/IVAC方案交替治疗各2个疗程（共4个疗程）。

初诊除外CNS受累的BL患者的治疗方案中均应包含CNS预防策略，尤其是选择不含有可以穿透血脑屏障药物的R-DA-EPOCH方案的患者，推荐进行腰椎穿刺鞘内注射预防CNS复发。对于初诊合并CNS受累（脑脊液和脑实质）的BL患者，不推荐选择R-DA-EPOCH方案治疗（推荐强度B，证据强度2b）。一项回顾性研究纳入120例基线CNS受累（脑脊液和脑实质）患者，R-DA-EPOCH方案治疗后CNS复发的发生率（3年时达到35％）显著高于其他方案[25]。

（三）难治复发BL的治疗

**推荐意见：首次治疗结束后6～18个月内复发的BL患者预后极差，推荐进入临床研究。首次治疗结束18个月后复发患者，可推荐参与临床研究或接受强剂量化疗。T细胞重定向疗法［嵌合抗原受体T（CAR-T）细胞或双特异性抗体］也可考虑在难治复发BL患者中应用。**

对于首次治疗结束后6～18个月内复发的BL患者，推荐进入临床研究。首次治疗结束18个月后复发患者，可推荐参与临床研究或接受强剂量化疗，如RICE、RIVAC及RGDP方案等，并序贯自体造血干细胞移植（auto-HSCT）或异基因造血干细胞移植（allo-HSCT）（推荐强度B，证据强度2b）[26]–[27]，CAR-T细胞治疗（推荐强度B，证据强度2b）或格菲妥单抗联合维泊妥珠单抗治疗（推荐强度C，证据强度4）。

Short等[28]回顾性分析了38例难治复发BL患者的临床数据，显示难治复发BL患者预后极差，中位OS期为2.8个月，1年OS率为11％。其中早期（首次缓解时间<6个月）复发患者的中位OS期仅1.6个月，而晚期（首次缓解时间≥6个月）复发患者的中位OS期为5.0个月。大部分患者接受大剂量化疗，3例患者接受auto-HSCT，3例患者接受allo-HSCT，总反应率（ORR）仅为39％，晚期复发和早期复发患者的ORR分别为61％和0。

2025年，Samples等[29]报道了真实世界31例难治复发BL患者接受CD19 CAR-T细胞治疗的效果，治疗后1个月ORR为58％，CR率为42％，但治疗后6个月CR率降为19％。3级以上细胞因子释放综合征（CRS）发生率为65％，3级以上免疫效应细胞相关神经毒性综合征（ICANS）发生率为19％，28 d死亡率为16％。中位PFS期仅为2.3个月，中位OS期仅6.0个月。ZUMA-25研究首次评价商业化CD19 CAR-T细胞布瑞基奥仑赛（Brexu-cel）在≥1线系统性治疗后难治复发BL患者中的疗效。10例患者接受回输，ORR为100％，其中CR率为50％，中位PFS期及OS期分别为5.8个月和12.9个月。CR患者6个月PFS率及OS率分别为75％和100％。3级以上CRS及ICANS发生率分别为10％和60％[30]。另外，目前有3例难治性BL患者接受格菲妥单抗联合维泊妥珠单抗获得CR的病例报道[31]。提示以T细胞重定向疗法（CAR-T细胞或格菲妥单抗）为基础的治疗方案可推荐应用。

四、疗效评估及随访

**推荐意见：根据2014版Lugano淋巴瘤疗效评估标准进行定期疗效评估。**

推荐根据2014版Lugano淋巴瘤疗效评估标准进行疗效评估（表5）[11]。

**表5:** 2014版Lugano淋巴瘤疗效评估标准[11]

分类	基于PET-CT的标准	基于CT的标准
完全缓解	Deauville 5分评分系统（D5PS）评分1～3分，无新病灶，无骨髓浸润	靶病变（淋巴结或肿块）最大横截直径缩小至1.5 cm以下；无结外病变，肿大的器官缩小到正常大小，无新病灶，无骨髓浸润
部分缓解	D5PS评分4～5分，与基线相比摄取减低，无新病灶，与基线相比骨髓残留及摄取减低	与基线相比，最多6个靶病灶（最长直径×垂直于最长直径的短径）总和缩小>50％，肿大的脾脏较基线缩小>50％，无新病灶
病情稳定	D5PS评分4～5分，与基线相比病灶摄取无变化，无新病灶，骨髓受累无变化	与基线相比，最多6个靶病灶（最长直径×垂直于最长直径的短径）总和缩小<50％，无新病灶
病情进展	D5PS评分4～5分，与基线相比病灶摄取增加，淋巴结和骨髓新发或复发高氟代脱氧葡萄糖（FDG）摄取病灶	淋巴结最长直径>1.5 cm；靶病灶最长直径×最长直径的垂直径增长>50％，淋巴结直径增长（淋巴结<2 cm时增长0.5 cm；如果>2 cm，则增长1.0 cm）。新发或复发脾脏肿大淋巴结，或新发或复发骨髓浸润

对完成预定方案治疗的患者进行定期随访，前2年每3个月随访1次，随后3年每4～6个月随访1次，以后每年随访1次。随访内容包括病史、体格检查、血生化检查及影像学检查。影像学检查主要推荐颈/胸/腹/盆腔增强CT。
